# A Comparative Analysis of Self-Directed Learning and the Jigsaw Method in Medical Physiology Education

**DOI:** 10.7759/cureus.84986

**Published:** 2025-05-28

**Authors:** Pinaki Wani, Mayank Agarwal, Prabal Joshi

**Affiliations:** 1 Department of Physiology, All India Institute of Medical Sciences, Raebareli, IND

**Keywords:** active learning, jigsaw method, mbbs students, medical pedagogy, physiology education, self-directed learning

## Abstract

Background: Active learning strategies have become increasingly prominent in medical education. Among them, self-directed learning (SDL) and the Jigsaw method aim to enhance student engagement and improve knowledge retention. However, direct comparisons of these strategies within physiology education remain limited, particularly in the Indian context.

Objectives: This study aimed to compare the effectiveness of SDL and the Jigsaw method on selected physiology topics among first-year Bachelor of Medicine and Bachelor of Surgery (MBBS) students.

Methods: We conducted a quasi-experimental crossover study at the All India Institute of Medical Sciences, Raebareli, India, between February and March 2025. First-year MBBS students participated in both SDL and Jigsaw sessions covering two different topics. In Phase 1, students from Batch A engaged in SDL, while Batch B employed the Jigsaw method to learn about ascending tracts. In Phase 2, the methods were reversed: Batch A used the Jigsaw approach, and Batch B engaged in SDL to study sleep physiology. Each session lasted 150 minutes. Knowledge gain was assessed using presession and postsession multiple-choice question (MCQ) tests. Students' perceptions regarding their learning experience, engagement, autonomy, and knowledge retention were measured using a validated 5-point Likert scale questionnaire. The Wilcoxon signed-rank test was used for statistical analysis, with a p value of less than 0.05 considered statistically significant.

Results: A total of 88 students underwent crossover. Both SDL and Jigsaw sessions significantly improved posttest MCQ scores compared to pretest scores. The two methods showed no significant difference in the overall pretest and posttest scores. However, students rated the Jigsaw method higher than SDL for overall experience (median 4.5 vs. 3.5; p < 0.001), engagement (4.7 vs. 4; p < 0.001), and knowledge retention (4.3 vs. 4; p < 0.001). Autonomy scores did not differ significantly (4.5 vs. 4.5; p = 0.16). A majority (69.3%) preferred the Jigsaw approach over SDL.

Conclusion: Both the SDL and Jigsaw methods effectively enhance learning outcomes in selected physiology topics among first-year MBBS students. However, the Jigsaw method demonstrates superior overall experience, student engagement, and perceived knowledge acquisition and retention.

## Introduction

The landscape of medical education has witnessed a fundamental shift from traditional teacher-centered instruction to more student-centered and active learning methodologies [1,2]. Traditional lecture-based teaching, although efficient for delivering large volumes of information, often results in passive learning and limited student engagement. On the other hand, active learning strategies have consistently shown better academic performance, long-term retention, and improved student motivation [3-5]. The shift toward competency-based medical education (CBME) in India further reinforces the need for pedagogical innovations that promote critical thinking, collaboration, and lifelong learning [2]. In this context, strategies such as self-directed learning (SDL) and the Jigsaw technique have garnered increasing attention [4,5]. Both approaches are grounded in adult learning principles and constructivist theories, yet they differ markedly in their implementation and underlying philosophy [6]. Social constructivism posits that knowledge emerges from social interactions and collaboration, as illustrated by methods such as the Jigsaw approach. In contrast, radical constructivism emphasizes that individuals form knowledge through their own understanding, as exemplified in SDL. These methodologies contribute to andragogy or cognitive constructivism, which asserts that knowledge is built through mental processes such as attention, perception, motivation, and learning orientation.

SDL has emerged as one of the strategies in the CBME framework [2]. SDL is a process in which individuals proactively assess their learning needs, set specific goals, determine the necessary resources for learning, select appropriate learning strategies, and evaluate the outcomes of their learning efforts, either autonomously or with limited facilitative guidance. This approach places primary responsibility for the learning endeavor directly on the individual. At its core, SDL is characterized by learner autonomy, initiative, personal responsibility, and intrinsic motivation [4,7]. This method is particularly relevant in medical education, where lifelong learning is essential due to the rapid advancement of clinical knowledge [8].

In contrast to the individualistic nature of SDL, the Jigsaw method is a cooperative learning technique that fosters student interdependence. In this method, students are initially divided into "home groups," and each member is assigned a unique subtopic related to the overall learning objective. Subsequently, students with the same subtopic from different home groups come together to form "expert groups." Within these expert groups, students collaborate to deepen their understanding of their assigned subtopic through focused discussion and sharing insights. Once the expert group members have comprehensively understood their subtopic, they return to their original home groups. In this phase, each student takes on the role of an expert, teaching their subtopic to their peers within the home group. This process ensures that each home group member gains an understanding of all the subtopics, contributing to the group's overall comprehension of the main topic. The Jigsaw method is characterized by this structured collaborative learning process, where individual accountability for mastering a subtopic and group interdependence for achieving a common learning goal is paramount. This approach encourages peer teaching, active participation, responsibility, and interdependence, boosting students' comprehension and critical thinking skills [5,9].

Physiology is a core subject in the first-year Bachelor of Medicine and Bachelor of Surgery (MBBS) curriculum, forming the basis for understanding clinical medicine [10]. However, its abstract concepts and complex mechanisms may challenge novice learners, particularly when taught passively. Active learning strategies, such as SDL and the Jigsaw method, address these challenges by fostering more profound understanding, student engagement, and collaborative skills [4,5,7,9]. Despite growing evidence of the individual effectiveness of these methods in medical physiology, direct comparative studies, particularly in the context of Indian undergraduate medical education, are scarce.

A few studies have compared Jigsaw and SDL with traditional teaching-learning methods; however, direct comparisons between SDL and the Jigsaw technique are notably lacking [11-14]. To the best of our knowledge, this is the first study to compare SDL with the Jigsaw method in physiology education in India.

This study aims to compare the relative effectiveness and perceptions of SDL and Jigsaw methods in a crossover format for selected physiology topics among first-year MBBS students. The efficacy of both approaches was evaluated using pre- and postintervention multiple-choice question (MCQ) scores. Additionally, students' perceptions were gathered through a structured questionnaire to assess their learning experiences and preferences. The findings of this study may guide the effective integration of these active learning strategies into preclinical medical education.

## Materials and methods

Study design and setting

This quasi-experimental crossover study was conducted between February 2025 and March 2025 at the Department of Physiology, All India Institute of Medical Sciences, Raebareli, India. Ethical clearance was obtained before the study's commencement, and all students' participation was voluntary. Digital informed consent via Google Forms (Google LLC, Mountain View, CA) was obtained from each participant.

Participants and sample size

The study involved first-year MBBS students from the 2024-2025 academic batch. Using Cochran's formula for proportion, the necessary sample size was calculated. The formula is n = N / (1 + N * MOE^2^), where N is the total number of medical students (100) in the 2024-2025 batch, MOE is the margin of error (0.05), and n is the desired sample size. It was determined that at least 80 responses were needed from a class of 100 to ensure an MOE within 5% at a 95% confidence level.

Development of study tools

The authors selected the topics "ascending tracts" and "sleep physiology" for the Jigsaw and SDL activities, as these topics had been recently covered in lectures. The first author initially drafted the pretest and posttest MCQs, which were subsequently reviewed and validated by all three authors. Likewise, the first author prepared the questionnaire, and all authors validated its content.

A total of 10 pretest and 10 posttest MCQs were finalized for each topic. Each MCQ had three distractors and one correct option. There was no negative marking for incorrect responses. The MCQs reflected varied levels of Bloom's taxonomy (see the Appendix). The MCQs were displayed on the screen with a one-minute timer for each question via PowerPoint (Microsoft Corp., Redmond, WA). Students submitted their responses through Google Forms (Google LLC), accessed via a quick-response code as was used in a previous study [15].

The questionnaire used a 5-point Likert scale and consisted of four sections. These included overall experience, engagement and participation, autonomy, and knowledge acquisition and retention. Additionally, it included one open-ended question that invited students to provide any further feedback they wished to share, as well as one closed-ended question asking about their overall preference between the Jigsaw and SDL.

Implementation

The students were informed about the scheduled dates for the Jigsaw and SDL sessions in advance. They received an overview of both methodologies; however, the specific topics were not disclosed. Students were instructed to bring their textbooks and mobile phones on the assigned days. The study was conducted in two phases in the Department of Physiology. Instructors monitored the sessions but did not intervene in the students' learning process. Students were not assisted in either method by the instructors.

Phase 1 focused on the topic "ascending tracts," which was divided into five subtopics that were the dorsal column-medial lemniscus system, the spinothalamic tract, the spinocerebellar tract, the analgesia system, and the applied aspects of ascending tracts. These subtopics formed the basis of five expert groups. Out of 100 enrolled students, 94 were present during Phase 1. Students with roll numbers 1-50 were assigned to Batch A, and those with roll numbers 51-100 were assigned to Batch B. Students of Batch B, arranged in ascending order of roll number, were assigned to the Jigsaw method, with home groups formed by grouping every five consecutive students. Students from Batch B who could not form a complete group of five were shifted to SDL, along with all students from Batch A. In total, nine jigsaw home groups comprising 45 students from Batch B were formed. The remaining 49 students participated in SDL.

**Figure 1:**
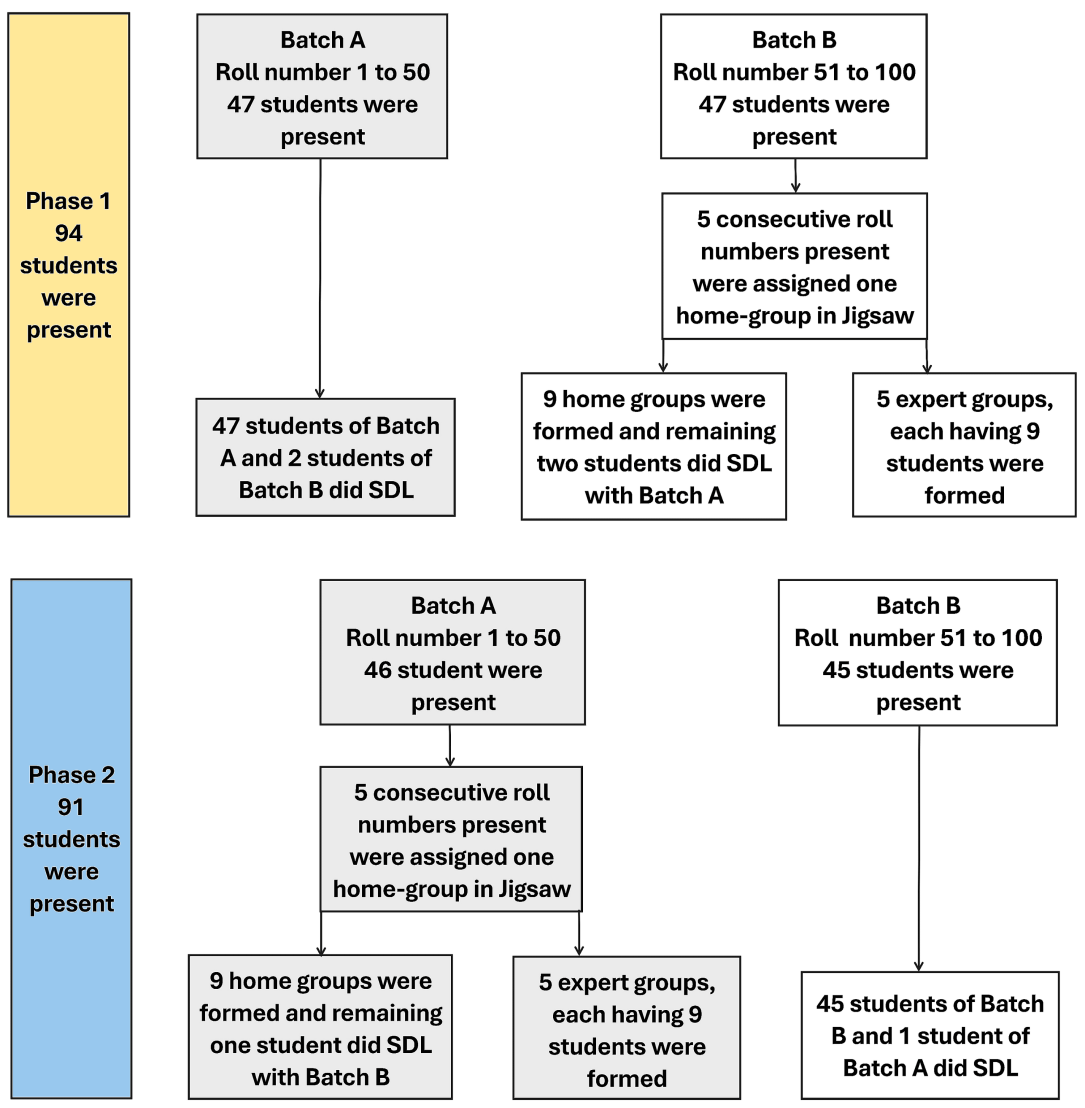
Flowchart illustrating the implementation of the study method SDL, self-directed learning

Phase 2 was conducted one week later and addressed the topic of "sleep physiology," which was also divided into five subtopics that were nonrandom eye movement sleep, random eye movement sleep, circadian rhythm and melatonin, electroencephalogram (EEG), and applied aspects of sleep and EEG. These subtopics formed the basis of five expert groups. A crossover design was implemented in this phase. This crossover ensured that all students experienced both Jigsaw and SDL methods, thereby reducing individual learning variability and enhancing internal validity. Out of 100 students, 91 were present in Phase 2. Students from Batch A, in ascending order and grouped in multiples of five, participated in the Jigsaw method. This formed nine home groups comprising 45 students. The remaining students of Batch A, along with all students from Batch B, participated in SDL. Figure 1 presents a simplified flowchart of the implementation process.

Pretest and posttest MCQ assessments were administered immediately before and after the SDL and Jigsaw sessions in both phases. The total duration of the Jigsaw and SDL in both phases was 150 minutes. After completing both phases, students were given an anonymous structured questionnaire in Google Forms (Google LLC) to gather their perceptions of the two learning techniques. Marks of the pretests and posttests for both phases were not disclosed to students before they responded to the questionnaire.

Statistical analysis

Upon analysis, it was observed that one student from each of Batch A and Batch B was absent in both phases and was, therefore, excluded from the study. Among the remaining participants, 44 students from Batch A who underwent SDL in Phase 1 subsequently participated in the Jigsaw method in Phase 2. Conversely, 44 students from Batch B who were exposed to the Jigsaw method in Phase 1 engaged in SDL during Phase 2. To preserve the integrity of the crossover design and ensure balanced exposure to both instructional methods, only these 88 students (44 from each batch) who completed both phases were included in the final analysis.

Data entry was conducted using Microsoft Excel 365 (Microsoft Corp.). For statistical analysis, the IBM Statistical Package for the Social Sciences Statistics for Windows, version 27 (released 2020; IBM Corporation, Armonk, NY) was utilized. Results are shown as mean ± standard deviation, median (interquartile range, IQR), and proportions (frequencies and percentages). Analysis of variance (ANOVA) with post hoc Tukey's test compared pretest and posttest scores for batches A and B in both phases. Repeated measures ANOVA with Bonferroni correction was employed to evaluate pretest and posttest scores within the same batch across both phases. Levene's test for equality of variances and Mauchly's test of sphericity were performed prior to one-way ANOVA and repeated measures ANOVA to assess the homogeneity of variances. The Wilcoxon signed-rank test was used to assess questionnaire scores. Cronbach's alpha tested the internal reliability of the questionnaire. A p value of less than 0.05 was deemed statistically significant.

## Results

Of the 100 students in the cohort, 88 (44 students from each of the two batches, A and B) completed all four sessions and were included in the analysis. Batch A included students from roll numbers 1-50, while Batch B included students from roll numbers 51-100.

Tables 1, 2 show the mean scores of the pretest and posttest obtained by Batches A and B in both phases. Both learning methods were effective, as indicated by the significant increase in posttest scores compared to pretest scores in both batches. There was no difference in the SDL and Jigsaw pretest scores among the same batches, indicating that the MCQs in both phases were comparable in difficulty for the same batch. However, the posttest score of the Jigsaw was significantly lower compared to the posttest score of SDL for Batch A. In contrast, the posttest score of the SDL was significantly lower than that of the Jigsaw for Batch B. The results indicate that the posttest scores in Phase 2 for both batches were significantly lower than those in Phase 1. This decline occurred regardless of the instructional method applied in Phase 2.

**Table 1:** Comparison of the pretest and posttest mean scores obtained by Batch A in both phases ANOVA, analysis of variance; SDL, self-directed learning

SDL		Jigsaw		p (repeated measure of ANOVA with Bonferroni correction)
Pretest phase 1	Posttest phase 1	Pretest phase 2	Posttest phase 2	
5.2 ± 2.1	7.4 ± 1.2	4.7 ± 1.4	5.7 ± 1.9	SDL pretest vs. posttest: <0.001
				Jigsaw pretest vs. posttest: 0.014
				SDL vs. Jigsaw posttest: <0.001
				SDL vs. Jigsaw pretest: 1.000

**Table 2:** Comparison of the pretest and posttest mean scores obtained by Batch B in both phases ANOVA, analysis of variance; SDL, self-directed learning

Jigsaw		SDL		p (repeated measure of ANOVA with Bonferroni correction)
Pretest phase 1	Posttest phase 1	Pretest phase 2	Posttest phase 2	
5.2 ± 2.1	7.7 ± 1.6	4.5 ± 1.6	5.8 ± 1.4	Jigsaw pretest vs. posttest: <0.001
				SDL pretest vs. posttest: 0.004
				SDL vs. Jigsaw posttest: <0.001
				SDL vs. Jigsaw pretest: 0.484

ANOVA followed by post hoc Tukey analysis revealed no significant difference in the posttest scores between Batches A and B in Phase 1 (p = 0.992) and Phase 2 (p = 1.000), suggesting comparable effectiveness of both learning techniques. However, the posttest score of Batch A in Phase 1 (SDL) was significantly higher (p < 0.001) than that of Batch B in Phase 2 (SDL). Likewise, the posttest score of Batch B in Phase 1 (Jigsaw) was significantly higher (p < 0.001) than that of Batch A in Phase 2 (Jigsaw). These findings indicate that both batches performed better in Phase 1 than in Phase 2.

Pretest scores showed no significant difference between Batches A and B in Phase 1 (p = 1.000) and Phase 2 (p = 0.860). Furthermore, no significant difference was observed between the pretest score of Batch A in Phase 1 (SDL) and Batch B in Phase 2 (SDL) (p = 0.466) or between the pretest score of Batch B in Phase 1 (Jigsaw) and Batch A in Phase 2 (Jigsaw) (p = 0.998). Thus, pretest MCQs were equally difficult for both batches in both phases. The questionnaire demonstrated high internal reliability, as assessed by a Cronbach's alpha of 0.900.

Table 3 presents the students' responses to the questionnaire on a 5-point Likert scale, along with a comparison of the median (IQR) scores for each question between the Jigsaw and SDL sessions. Students rated the Jigsaw method higher than SDL for most of the questions. Of the maximum possible score of 50 for 10 questions on the Likert scale, the mean Jigsaw score was 42.99 ± 6.28, while the mean SDL score was 38.41 ± 7.35, with a p value less than 0.001 and an effect size of 0.46 in the Wilcoxon signed-rank test.

**Table 3:** Responses of students to the questionnaire IQR, interquartile range; SD, standard deviation; SDL, self-directed learning

Questions with subsections	Frequency (percentage) of Likert scale responses					Median (IQR)	Mean ± SD	p (Wilcoxon signed-rank test)
	1	2	3	4	5			
Overall experience								
Overall, the Jigsaw method facilitated my learning effectively	0 (0%)	2 (2.3%)	18 (20.5%)	21 (23.9%)	47 (53.4%)	5 (1)	4.28 ± 0.87	<0.001
Overall, the SDL method facilitated my learning effectively	1 (1.1%)	4 (4.5%)	26 (29.5%)	41 (46.6%)	16 (18.2%)	4 (1)	3.76 ± 0.84	
I could adapt easily to the Jigsaw method	0 (0%)	4 (4.5%)	17 (19.3%)	34 (38.6%)	33 (37.5%)	4 (1)	4.09 ± 0.87	0.265
I could adapt easily to the SDL method	2 (2.3%)	4 (4.5%)	20 (22.7%)	36 (40.9%)	26 (29.5%)	4 (2)	3.91 ± 0.96	
Engagement								
I felt motivated to learn during the Jigsaw session	0 (0%)	2 (2.3%)	11 (12.5%)	26 (29.5%)	49 (55.7%)	5 (1)	4.39 ± 0.79	<0.001
I felt motivated to learn during the SDL session	1 (1.1%)	7 (8%)	22 (25%)	34 (38.6%)	24 (27.3%)	4 (2)	3.83 ± 0.96	
I found the Jigsaw method engaging	0 (0%)	0 (0%)	10 (11.4%)	27 (30.7%)	51 (58%)	5 (1)	4.47 ± 0.69	<0.001
I found the SDL method engaging	2 (2.3%)	15 (17%)	35 (39.8%)	25 (28.4%)	11 (12.5%)	3 (1)	3.32 ± 0.98	
I actively learned during the Jigsaw session	0 (0%)	0 (0%)	10 (11.4%)	27 (30.7%)	51 (58%)	5 (1)	4.47 ± 0.69	<0.001
I actively learned during the SDL session	0 (0%)	8 (9.1%)	25 (28.4%)	29 (33.3%)	26 (29.5%)	4 (2)	3.83 ± 0.96	
Autonomy								
The Jigsaw method encouraged me to take control of my learning process	1 (1.1%)	4 (4.5%)	14 (15.9%)	28 (31.8%)	41 (46.6%)	4 (1)	4.18 ± 0.94	0.114
The SDL method encouraged me to take control of my learning process	2 (2.3%)	3 (3.4%)	21 (23.9%)	31 (35.2%)	31 (35.2%)	4 (2)	3.98 ± 0.97	
The Jigsaw method allowed me to explore topics efficiently	0 (0%)	3 (3.4%)	5 (5.7%)	31 (35.2%)	49 (55.7%)	5 (1)	4.43 ± 0.76	0.386
The SDL method allowed me to explore topics efficiently	0 (0%)	2 (2.3%)	14 (15.9%)	24 (27.3%)	48 (54.5%)	5 (1)	4.34 ± 0.83	
Knowledge acquisition and retention								
I feel confident in my understanding of the physiology topics covered in the Jigsaw method	0 (0%)	4 (4.5%)	15 (17%)	31 (35.2%)	38 (43.2%)	4 (1)	4.17 ± 0.87	0.027
I feel confident in my understanding of the physiology topics covered in the SDL method	0 (0%)	6 (6.8%)	23 (26.1%)	34 (38.6%)	25 (28.4%)	4 (2)	3.89 ± 0.90	
The Jigsaw method helped me retain the information learned effectively	0 (0%)	3 (3.4%)	15 (17%)	31 (35.2%)	39 (44.3%)	4 (1)	4.21 ± 0.85	0.010
The SDL method helped me retain the information learned effectively	0 (0%)	6 (6.8%)	26 (29.5%)	31 (35.2%)	25 (28.4%)	4 (2)	3.85 ± 0.92	
The Jigsaw method encouraged critical and analytical thinking	0 (0%)	1 (1.1%)	9 (10.2%)	40 (45.5%)	38 (43.2%)	4 (1)	4.31 ± 0.70	<0.001
The SDL method encouraged critical and analytical thinking	1 (1.1%)	7 (8%)	30 (34.1%)	29 (33%)	21 (23.9%)	4 (1)	3.71 ± 0.96	

Table 4 shows the overall mean and median scores for the four subsections of the questionnaire. Except for autonomy, students significantly favored the Jigsaw method over SDL for engagement, knowledge acquisition and retention, and overall experience.

**Table 4:** Mean and median scores of questionnaire subsections IQR, interquartile range; SD, standard deviation; SDL, self-directed learning

Questionnaire subsections	Jigsaw		SDL		p (Wilcoxon signed-rank test)
	Median (IQR)	Mean ± SD	Median (IQR)	Mean ± SD	
Overall experience	4.5 (1)	4.38 ± 0.72	3.5 (1)	3.54 ± 0.85	<0.001
Engagement	4.7 (1)	4.31 ± 0.67	4 (1.3)	3.86 ± 0.83	<0.001
Autonomy	4.5 (1)	4.31 ± 0.73	4.5 (1.5)	4.16 ± 0.81	0.163
Knowledge acquisition and retention	4.3 (1.7)	4.23 ± 0.69	4 (1)	3.81 ± 0.83	<0.001

**Figure 2:**
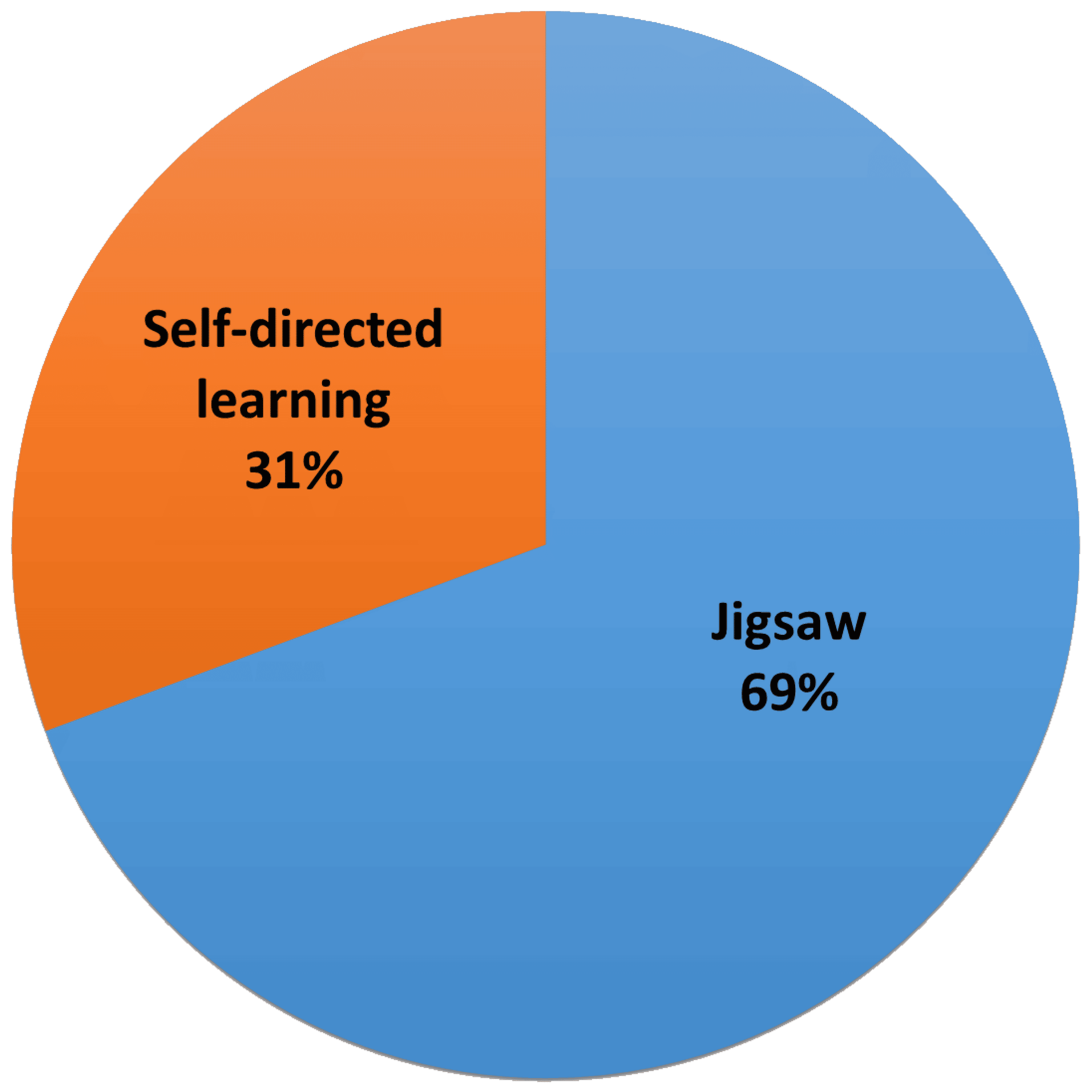
Student's responses to the preferred learning method

Further, when asked about their preferred learning method, a majority of students (61 out of 88) reported favoring the Jigsaw technique over SDL. Only 27 students expressed a preference for SDL, as shown in Figure 2.

In open-ended responses, the majority (41, 46.6%) either responded that both methods were good or that there were no suggestions. Several students suggested incorporating group discussions at the end of SDL to resolve doubts (9, 10.2%). A few students responded that ensuring equal participation from all students (11, 12.5%) and allowing students to select their groups could enhance engagement (3, 3.4%) in the Jigsaw method. Combining SDL for self-study and jigsaw for group discussions was also recommended (3, 3.4%) to foster a more dynamic learning experience. In SDL, common issues included difficulty maintaining focus and motivation over long periods, feelings of boredom or fatigue (18, 20.5%), and challenges in understanding material without peer interaction or teacher involvement (3, 3.4%).

## Discussion

This quasi-experimental crossover study aimed to compare the effectiveness of the SDL and Jigsaw methods in selected physiology topics among first-year MBBS students. The MCQ results indicate that there was no significant difference in posttest scores between the two instructional strategies. However, a closed-ended question and overall questionnaire score revealed a clear preference among students for the Jigsaw method over SDL.

The improvement in posttest scores following both SDL and Jigsaw sessions confirms the effectiveness of active learning strategies in enhancing students' academic performance. This finding aligns with existing literature, which emphasizes the superiority of interactive, student-centered approaches over didactic lectures in promoting knowledge acquisition and retention among medical undergraduates [3-5]. However, the pattern of results reveals notable differences in the impact of these methods across phases and batches.

A significant observation was that posttest scores were consistently higher in Phase 1, regardless of the learning strategy used. The likely explanation for this trend can be attributed to higher initial motivation to perform better during their initial exposure to a new instructional method, cognitive freshness, and possibly lower academic fatigue during Phase 1 of employing learning methods. The nature of the topic could have influenced students' performance more than the teaching method. "Ascending tracts" follow structured pathways, making learning easier. Furthermore, this topic is also covered in anatomy. We perceive that sleep physiology is more abstract, requiring higher cognitive integration, which neither method facilitated effectively. These explanations are merely speculative, and further studies are needed for their confirmation.

When comparing the two methods based on students' questionnaire responses, both methods were perceived as equally adaptable to the students. The Jigsaw method consistently demonstrated higher levels of student engagement, motivation, perceived learning, comprehension, knowledge retention, and critical thinking. Our findings are similar to those of a few other studies that aimed to investigate the effects of Jigsaw on first-year MBBS students in physiology education [13,14,16]. Soundariya et al. [13] assessed the effectiveness of the Jigsaw technique as an active learning strategy in teaching physiology to first-year MBBS students. The findings indicated enhanced student engagement, improved communication skills, and a better understanding of concepts. However, challenges included time constraints and adaptation difficulties, suggesting limited routine application. The study by Bhandari et al. [14] evaluated the Jigsaw method as a cooperative learning strategy for first-semester MBBS students studying physiology. Feedback indicated that the method enhanced peer interaction, comprehension, and communication skills, suggesting its effectiveness in medical education. The study by Bansal et al. [16] assessed the effectiveness of the modified Jigsaw technique in teaching physiology to first-year MBBS students. Feedback indicated enhanced engagement, better understanding, and improved communication skills. Students expressed a preference for more such sessions.

The Jigsaw method offers several claimed benefits in the educational context. It enhances student engagement through active participation in both expert and home groups, as well as through peer interaction during the teaching phase. By requiring students to become experts on a specific subtopic and then teach it to their peers, the Jigsaw method promotes deeper knowledge acquisition and improved retention of information. The processes of analyzing, synthesizing, and explaining information within expert groups and then articulating it to home groups contribute significantly to the development of critical thinking skills. Furthermore, the Jigsaw method inherently fosters collaborative skills as students work together in both expert and home groups to achieve a common learning goal. Communication skills are also honed as students explain their subtopics and engage in discussions with their peers. Ultimately, the Jigsaw method fosters a sense of responsibility for both individual learning and the collective learning of group members, as each student's contribution is crucial to the group's success [5,9,17,18].

Despite its numerous advantages, the Jigsaw method also presents potential challenges and limitations. The implementation of the Jigsaw method can be time-consuming, requiring adequate time for expert group meetings, teaching within home groups, and subsequent synthesis activities. There is also the possibility of uneven participation among group members, with some students potentially relying more on others or not fully engaging with their assigned subtopic. The effectiveness of the Jigsaw method is also dependent on the expertise and teaching abilities of peers, which can vary in quality and might not always be as comprehensive or accurate as instruction from an educator. Furthermore, the complexity of the Jigsaw method, with its multiple stages and group interactions, might lead to working memory overload or distractions for some students [17,18].

SDL was rated equal to Jigsaw in promoting learner autonomy. These findings suggest that integrating either strategy can effectively foster autonomy among undergraduate medical students. However, students perceived SDL as inferior to the Jigsaw method. Contrary to our results, several Indian studies have reported high student satisfaction with SDL in physiology education [11,12]. The study by Bhandari et al. [11] introduced SDL in physiology for first-year MBBS students. Postsession feedback indicated increased student preparedness and awareness of learning strengths. However, some students reported limited improvement in analytical skills. Both students and faculty expressed satisfaction with SDL implementation. The study by Nandini et al. [12] compared SDL with traditional lectures in teaching physiology to first-year MBBS students. Findings revealed that SDL sessions led to higher posttest scores and improved student engagement. Most students favored SDL, citing enhanced autonomy and goal setting, indicating its effectiveness in medical education. However, it should be noted that in these studies, either only SDL sessions were conducted [11] or SDL was compared with traditional teaching methods [12]. In support of our study results, several studies have also noted the relative lack of readiness for SDL among Indian medical undergraduates [19,20]. The study by Kiran and Hema [19] assessed SDL readiness among second- and third-year MBBS students. Results showed that 56.8% had moderate readiness, while only 42.9% demonstrated high readiness. The study by Premkumar et al. [20] assessed SDL readiness among Indian medical students across different academic years. Findings revealed a significant decline in readiness scores as students progressed through their training. Qualitative insights highlighted the influence of curriculum structure and cultural factors on SDL readiness. The study recommends curricular reforms to better support and enhance SDL among medical students.

SDL offers numerous benefits. It actively engages students by allowing them to learn at their own pace, tailored to their individual needs. This personalized approach nurtures a genuine interest in learning and promotes intrinsic motivation. As a result, learners develop into lifelong seekers of knowledge. The core processes of SDL involve identifying learning needs, setting goals, and evaluating outcomes, all of which play a crucial role in enhancing critical thinking skills. Additionally, SDL provides flexibility regarding time, pace, and learning strategies. This adaptability makes education more accessible and convenient, accommodating various individual circumstances and learning preferences [4,21,22].

Despite its numerous advantages, SDL also presents potential challenges and limitations. It necessitates high levels of self-discipline and motivation, which may not be uniformly present across all learners. Individuals new to a subject may encounter difficulties in identifying appropriate learning goals and resources, which can lead to inefficient learning or the overlooking of crucial foundational knowledge. Moreover, SDL might not be universally suitable. The relative lack of structure in SDL may pose a challenge for novice learners, particularly those in the early stages of medical education, who often require more guidance and scaffolding to navigate complex topics [19-22].

An important pedagogical implication arising from the findings is the potential benefit of blending these two strategies. A theoretical model for integrating SDL and Jigsaw could involve students first undergoing SDL, independently studying the assigned topic. This should be followed by the Jigsaw, in which expert groups have already completed SDL and are thus more prepared to share knowledge among themselves. While SDL cultivates independence and lifelong learning habits, the Jigsaw method enhances communication, teamwork, and collaborative reasoning. An integrated model that sequentially employs SDL to promote independent preparation, followed by Jigsaw sessions to consolidate understanding through peer teaching, may yield optimal outcomes. Allowing students to choose their own group members may enhance the impact of Jigsaw.

Limitations

The short interval between the phases might have introduced fatigue, which could explain the lower posttest scores in Phase 2. The single-center design restricts generalizability across institutions. Additionally, the study focused only on two physiology topics: broader generalizability would require replication across more subjects and topics of varying complexity. Another consideration is the subjective nature of the perception questionnaire. While it provides valuable insights into learner satisfaction and engagement, it does not objectively measure the depth of learning or long-term retention. Likert scale responses could be affected by recency bias, especially in educational studies. The Hawthorne effect, that is, a change in student behavior in the instructor's presence, could be a limiting factor, but a crossover study design limits its impact on results. Future studies may include delayed posttests or performance-based assessments to evaluate lasting impacts. Moreover, qualitative methods such as focus group discussions may add depth to understanding students' experiences and preferences.

## Conclusions

Both the SDL and Jigsaw methods effectively enhance learning outcomes in the field of physiology. However, the Jigsaw method has a distinct advantage in promoting engagement, knowledge acquisition, retention, and overall student satisfaction. These findings support its inclusion as a recommended component in the active learning toolkit for preclinical medical education. At the same time, fostering SDL skills remains essential for developing lifelong learners. A judicious integration of both methods, tailored to the complexity of the topic and the learner's readiness, may offer the most pedagogically sound approach within the CBME framework. The lack of long-term outcome data and the potential influence of phasewise motivation drop warrants the external validity of the present study findings.

## Appendices

**Table 5:** MCQs used in the study and their cognitive-level classification according to the Bloom's taxonomy DCML, dorsal column-medial lemniscus; EEG, electroencephalogram; EMG, electromyogram; GABA, gamma-aminobutyric acid; GH, growth hormone; MCQs, multiple-choice questions; NMDA, N-methyl-D-aspartate; NREM, nonrapid eye movement; PGO, ponto-geniculo-occipital; RAS, reticular activating system; REM, rapid eye movement; RVM, rostral ventromedial medulla; SCN, suprachiasmatic nucleus; VPL, ventral posterolateral

MCQ	Option A	Option B	Option C	Option D	Cognitive level
Pretest day 1					
A research study investigates the role of neurotransmitters in the modulation of ascending pain pathways. Inhibiting which of the following neurotransmitters would most likely lead to increased pain perception	Substance P	Serotonin	Glutamate	GABA	Analyze
A 55-year-old man presents with a right-sided loss of fine touch, vibration, and proprioception below the T8 level, along with a left-sided loss of pain and temperature sensation at the same level. Which of the following is the most likely site of the lesion?	Right dorsal column at T8	Left spinothalamic tract at T8	Right hemisection of the spinal cord at T8	Left medial lemniscus at the level of the medulla	Apply
A patient experiences chronic pain but does not wake up during sleep, even when exposed to painful stimuli. Which of the following explains the underlying mechanism?	Thalamic relay neurons inhibit pain transmission during sleep	RAS modulates cortical perception of pain	The anterolateral system shuts down during deep sleep	The dorsal column pathway inhibits the spinothalamic tract	Understand
The descending analgesic system suppresses pain by	Inhibiting the nociceptive inputs directly in the thalamus	Modulating the release of excitatory neurotransmitters in the dorsal horn of the spinal cord	Activating C-fiber primary afferents to desensitize pain receptors	Enhancing the activity of the spinothalamic tract	Understand
In an experiment, stimulating the RVM decreases the firing rate of nociceptive dorsal horn neurons. Which neurotransmitter is most likely mediating this effect?	Glutamate	Serotonin	Substance P	Dopamine	Apply
A patient presents with impaired vibration and proprioception on the left side of the body below the T8 level. Where is the most likely site of the lesion?	Left dorsal column at the T8 level	Right medial lemniscus in the medulla	Left spinothalamic tract in the spinal cord	Right primary somatosensory cortex	Apply
A patient with a stroke affecting the right VPL nucleus of the thalamus presents with sensory deficits. Which of the following findings is most likely?	Loss of pain and temperature sensation on the right side of the body	Loss of vibration and proprioception on the left side of the body	Loss of vibration and proprioception on the right side of the body	Loss of crude touch and pain on the left side of the body	Apply
In an experimental model, a lesion is made in the anterior white commissure at the C5 spinal level. Which of the following sensory deficits would you expect?	Bilateral loss of proprioception below the C5 level	Ipsilateral loss of pain and temperature below the C5 level	Bilateral loss of pain and temperature at the C5 dermatome	Contralateral loss of vibration and fine touch below the C5 level	Apply
A patient undergoing an experimental pain modulation study receives a drug that blocks substance P receptors in the dorsal horn of the spinal cord. What is the expected physiological effect?	Decreased pain perception due to reduced nociceptive transmission	Increased pain sensitivity due to dorsal horn hyperexcitability	No change in pain perception, as substance P is unrelated to the spinothalamic tract	Increased proprioception due to enhanced dorsal column activity	Analyze
A 45-year-old patient presents with acute right upper quadrant pain that radiates to the right shoulder, suggesting gallbladder inflammation. What is the most likely mechanism responsible for the referred pain?	Convergence of visceral afferents from the gallbladder with somatic afferents from the diaphragm at the C3-C5 spinal level	Direct activation of spinothalamic tract neurons in the cervical spinal cord by gallbladder nociceptors	Spread of gallbladder inflammation to the cervical nerve roots via peritoneal irritation	Retrograde transmission of pain signals through the vagus nerve to the somatosensory cortex	Apply
Posttest day 1					
Which of the following best explains the impairment of two-point discrimination in dorsal column lesions?	Dorsal column carries fibers responsible for detecting changes in temperature and crude touch	Dorsal column integrates signals from multiple spinal segments to distinguish between closely spaced stimuli	Dorsal column transmits fine touch information that is processed in the somatosensory cortex for spatial resolution	Dorsal column inhibits spinothalamic pathways to enhance discriminatory touch	Understand
A patient with chronic pain is administered an experimental drug that selectively enhances enkephalin release in the dorsal horn. What is the most likely physiological outcome?	Decreased nociceptive transmission due to presynaptic inhibition of substance P release	Increased pain perception due to upregulation of NMDA receptor activity	No change in pain perception, as enkephalins only modulate muscle reflexes	Increased allodynia due to excitatory effects on second-order neurons	Analyze
Which of the following best explains the mechanism by which the descending analgesic system reduces pain perception?	Increasing GABAergic inhibition in the motor cortex	Facilitating the release of endorphins in the limbic system	Enhancing serotonin and norepinephrine release in the dorsal horn to activate inhibitory interneurons	Blocking prostaglandin synthesis in peripheral tissues	Understand
A 60-year-old patient presents with loss of proprioception and fine touch on the right side below the level of T8 but has intact pain and temperature sensation. Which of the following is the most likely site of the lesion?	Left medial lemniscus in the brainstem	Right dorsal column at T8	Left dorsal column at T8	Right ventroposterior nucleus of the thalamus	Apply
In an experiment, researchers found that patients with amputated limbs exhibit activation of the DCML pathway when the face is stimulated. What is the most plausible explanation?	Axonal regeneration of DCML neurons	Cortical reorganization of the primary somatosensory cortex	Compensatory activation of the spinothalamic tract	Selective degeneration of medial lemniscus fibers	Analyze
A patient is experiencing chronic pain despite no identifiable peripheral injury. Which of the following therapeutic interventions is most likely to enhance the activity of the descending analgesic system and provide relief?	Local anesthetic injection at the site of pain	Administering opioids that activate μ-receptors in the brainstem	Blocking NMDA receptors in the dorsal horn of the spinal cord	Surgical removal of the posterior horn	Apply
A patient with a spinal cord hemisection at T10 on the left side presents with sensory deficits. Which of the following findings would be expected?	Loss of pain and temperature sensation on the left below T10	Loss of proprioception and fine touch on the right below T10	Loss of pain and temperature sensation on the right below T10	Loss of pain and temperature on both sides below T10	Apply
A patient with a myocardial infarction reports pain radiating to the left arm. Which of the following best explains this phenomenon?	Direct activation of spinothalamic tract neurons by cardiac nociceptors	Convergence of visceral and somatic afferents onto common spinal neurons	Spread of inflammation from cardiac tissue to the brachial plexus	Activation of corticospinal fibers causing referred discomfort	Understand
In an experiment, a selective C-fiber blocker is applied to the spinal cord. Which of the following changes in pain perception is most likely?	Loss of sharp, well-localized pain	Complete loss of pain and temperature sensation	Loss of burning and aching pain, but retention of sharp pain	No change in pain perception due to redundancy in the spinothalamic tract	Apply
Which sensory deficits would a patient with a stroke in the left thalamic ventroposterior nucleus exhibit?	Loss of pain and temperature on the left side	Loss of fine touch and proprioception on the right side	Ipsilateral sensory loss affecting all modalities	No sensory deficits, as the thalamus processes only motor information	Apply
Pretest day 2					
A 52-year-old man with REM sleep behavior disorder undergoes a sleep study. Which EEG and EMG patterns are most likely observed during REM sleep?	Low-amplitude mixed-frequency EEG with muscle atonia	High-voltage delta waves with increased EMG activity	Low-voltage EEG with persistent muscle tone (absent atonia)	Sleep spindles and K-complexes with phasic muscle twitches	Apply
A 40-year-old patient undergoes polysomnography. During stable stage 3 NREM sleep, EEG shows high-amplitude delta waves. What is the most likely functional significance of this EEG pattern?	Active cortical processing and memory encoding	Synaptic downscaling and neural plasticity	Heightened sensory responsiveness to external stimuli	Increased sympathetic outflow and cardiovascular activation	Understand
A 15-year-old man undergoing an endocrinology evaluation shows deficient GH secretion. Which of the following sleep abnormalities is most likely contributing to this?	Reduced delta wave activity in stage 3 sleep	Increased REM sleep duration at the expense of NREM sleep	Fragmented sleep with frequent awakenings during REM sleep	Suppressed K-complex formation in stage 2 sleep	Apply
Which of the following best describes autonomic changes during REM sleep?	Predominant parasympathetic activity with stable heart rate and blood pressure	Alternating sympathetic and parasympathetic dominance with cardiovascular variability	Strong sympathetic activation leading to sustained hypertension	Complete autonomic suppression with minimal heart rate changes	Understand
A 25-year-old patient undergoes an eye-tracking study during REM sleep. Which of the following best describes the characteristics of REM-related eye movements?	Slow rolling movements controlled by the vestibular system	Rhythmic and stereotyped lateral movements driven by the oculomotor nucleus	Rapid, jerky, saccadic movements independent of external stimuli	Conjugate gaze shifts triggered by cortical activation	Remember
Which of the following best explains the physiological mechanism underlying REM sleep muscle atonia?	Increased activation of gamma motor neurons by the cerebellum	Excitation of corticospinal pathways leading to voluntary inhibition	Glycinergic and GABAergic inhibition of spinal motor neurons from the brainstem	Selective suppression of motor activity by the primary motor cortex	Understand
A patient with insomnia is prescribed exogenous melatonin. What is the primary mechanism by which melatonin promotes sleep?	It inhibits the ascending reticular activating system	It synchronizes the circadian clock by acting on SCN melatonin receptors	It enhances serotonin production, inducing drowsiness	It suppresses orexin neurons in the lateral hypothalamus	Understand
Which of the following physiological adaptations is most likely in a long-term night-shift worker who sleeps during the day?	Delayed cortisol peak in the evening instead of the morning	Increased melatonin secretion at the beginning of their work shift	Enhanced phase alignment with the natural light-dark cycle	Reduced exposure of the SCN to environmental cues	Apply
Which of the following cognitive functions is most negatively impacted by NREM sleep deprivation?	Emotional reactivity and fear processing	Attention and reaction time	Consolidation of procedural (motor) memory	Visual hallucinations and REM-related dream processing	Analyze
Which of the following best describes neurotransmitter activity during REM sleep?	Increased serotonin and norepinephrine drive cortical activation	High acetylcholine activity facilitates cortical desynchronization	Dopamine levels decrease, reducing dream vividness	Increased GABA in the pons suppresses REM-related eye movements	Remember
Posttest day 2					
An elderly patient undergoes a sleep study due to complaints of frequent nocturnal awakenings. Which of the following EEG changes is most likely in this patient compared to younger adults?	Increased REM sleep duration and enhanced slow-wave activity	Increased sleep spindles and prolonged NREM stage 3 sleep	Decreased slow-wave sleep and increased wake after sleep onset	Higher proportion of stage 3 NREM sleep relative to stage 2	Understand
A medical student stays awake for 36 hours. When allowed to sleep, their EEG shows a marked increase in slow-wave activity during NREM sleep. This change is most likely due to:	Increased activation of the suprachiasmatic nucleus	Reduced levels of adenosine in the basal forebrain	Homeostatic rebound in sleep pressure following deprivation	Increased gamma wave activity promoting sleep fragmentation	Analyze
Which of the following neurotransmitter changes is most crucial for initiating NREM sleep?	Increased norepinephrine release from the locus coeruleus	Enhanced GABAergic inhibition from the ventrolateral preoptic nucleus	Increased histaminergic activity from the tuberomammillary nucleus	Enhanced dopamine signaling from the substantia nigra	Remember
A 32-year-old patient undergoes polysomnography. Which of the following states most closely resembles EEG findings during REM sleep?	Deep NREM sleep (stage 3)	Quiet wakefulness with alpha rhythms	Active wakefulness with desynchronized beta waves	Drowsiness with increased theta wave activity	Understand
A student preparing for a music performance has a disrupted sleep pattern. Which aspect of memory consolidation is most likely to be impaired due to loss of REM sleep?	Declarative memory formation (e.g., factual knowledge)	Procedural memory enhancement (e.g., motor skill learning)	Semantic memory recall (e.g., word associations)	Episodic memory encoding (e.g., personal experiences)	Apply
Which of the following best describes the functional significance of PGO waves during REM sleep?	They synchronize hippocampal activity for memory consolidation	They trigger motor activation and voluntary movements	They contribute to dream generation and rapid eye movements	They inhibit sensory input from the thalamus to prevent arousal	Understand
Which of the following best describes the role of the suprachiasmatic nucleus in circadian rhythm regulation?	It generates circadian rhythms independently but requires retinal input for synchronization	It primarily controls sleep onset by directly inhibiting the thalamus	It regulates circadian cycles only in the presence of external light cues	It stimulates the pineal gland to produce melatonin in response to light exposure	Understand
A 56-year-old airline pilot frequently travels across multiple time zones. Exposure to bright light at night will most likely have which effect on melatonin secretion	Increase in melatonin release due to suprachiasmatic nucleus activation	Decrease in melatonin release due to inhibition of the pineal gland	No change in melatonin levels since secretion is independent of light	Irregular melatonin pulses due to direct retinal ganglion cell activation	Apply
Which of the following best describes the relationship between the circadian rhythm and cortisol secretion?	Cortisol secretion is highest at night and lowest in the early morning	Cortisol levels peak in the early morning and decline throughout the day	The suprachiasmatic nucleus directly stimulates adrenal cortisol release during REM sleep	Cortisol follows an ultradian rhythm independent of the circadian cycle	Remember
Elderly individuals often experience circadian rhythm alterations, including which of the following changes	Increased melatonin secretion at night	Delayed sleep phase with later sleep onset	Advanced sleep phase with earlier sleep onset and wake times	Increased ability to adapt to time zone changes	Understand
